# Synthesis and Manipulation of Single-Crystalline Lithium Nickel Manganese Cobalt Oxide Cathodes: A Review of Growth Mechanism

**DOI:** 10.3389/fchem.2020.00747

**Published:** 2020-09-09

**Authors:** Ting Wang, Keliang Ren, Miao He, Wenhao Dong, Wei Xiao, Hongyu Pan, Jia Yang, Yang Yang, Ping Liu, Zhijie Cao, Xiaobo Ma, Hailong Wang

**Affiliations:** ^1^Advanced Energy Storage Materials & Devices Lab, Ningxia University, Yinchuan, China; ^2^Ningxia Polytechnic, Yinchuan, China; ^3^Hubei Key Laboratory of Electrochemical Power Sources, College of Chemistry and Molecular Sciences, Wuhan University, Wuhan, China; ^4^Office of Frontier Technology, Ningxia Power and Energy Storage Lithium-Ion Battery Materials Engineering Technology Research Center, Zhongwei, China

**Keywords:** layered cathode, single-crystalline, growth mechanism, lithium ion batteries, particle morphology

## Abstract

Lithium nickel manganese cobalt oxide (NMC) cathodes are of great importance for the development of lithium ion batteries with high energy density. Currently, most commercially available NMC products are polycrystalline secondary particles, which are aggregated by anisotropic primary particles. Although the polycrystalline NMC particles have demonstrated large gravimetric capacity and good rate capabilities, the volumetric energy density, cycling stability as well as production adaptability are not satisfactory. Well-dispersed single-crystalline NMC is therefore proposed to be an alternative solution for further development of high-energy-density batteries. Various techniques have been explored to synthesize the single-crystalline NMC product, but the fundamental mechanisms behind these techniques are still fragmented and incoherent. In this manuscript, we start a journey from the fundamental crystal growth theory, compare the crystal growth of NMC among different techniques, and disclose the key factors governing the growth of single-crystalline NMC. We expect that the more generalized growth mechanism drawn from invaluable previous works could enhance the rational design and the synthesis of cathode materials with superior energy density.

## Introduction

Lithium nickel manganese cobalt oxide (NMC) cathodes have been critical pillars of advanced lithium ion batteries at current state (Chen et al., 2019; Xu et al., 2019; Zhou et al., 2019; Kim et al., 2020; Li et al., 2020; Wang et al., 2020b; Wu et al., 2020; Zhang, 2020; Zheng et al., 2020). The particle's crystallinity and morphology can put significant influences on the energy density, cycling stability, and rate capability of the NMC cathodes in practical applications (Liu et al., 2018; Fan et al., 2020). Most commercially available NMC are polycrystalline secondary particles aggregated by numerous nanosized primary particles, which usually maintain the spherical shape from the precursors. The polycrystalline nature of the secondary particles presents many issues, including (1) low tap density (<3.3 g cm^−3^) compared with the single-crystalline LiCoO_2_ cathode (3.9 g cm^−3^), which leads to an inadequate volumetric energy density (Kim et al., 2018), (2) the formation of microcracks inside the secondary particle incurred by the anisotropic lattice expansion and shrink during charging/discharging, which gradually deteriorates the cycling stability (Kim et al., 2018; Liu et al., 2018), and (3) the porous structure of the secondary particle prevents homogeneous carbon coating on each primary particle, which results in unsatisfactory electrical contact between the active material and the current collector (Kimijima et al., 2016b). In the recent decade, the chasing of larger gravimetric capacity has pushed the nickel content of the NMC to over 60%; as a tradeoff larger gravimetric capacity, these nickel-rich NMC cathodes are suffering more serious issues caused by the polycrystalline secondary particles (Dixit et al., 2017; Kim et al., 2018; Lee et al., 2018; Liang et al., 2018; Ryu et al., 2018; Fan et al., 2020; Wang et al., 2020b). The mandatory increase of the tap density to over 3.3 g cm^−3^ generally leads to rapid capacity fading since the polycrystalline secondary particles would crack and collapse during high-pressure electrode pressing (Kim et al., 2018). Although more Li^+^ ions can be extracted from Ni-rich cathodes, the electrostatic repulsion between oxygen slabs increases, too, which leads to a more drastic *c/a* ratio variation during electrochemical cycling. As a result of the anisotropic lattice volume change, the microcrack propagation is more serious in those Ni-rich NMC cathodes (Liu et al., 2018; Fan et al., 2020). Several strategies, such as concentration gradient structure and grain boundary coating, have been developed to improve the structural stability of the polycrystalline NMC cathode (Kim et al., 2018; Ouyang et al., 2018; Su et al., 2019; Lee et al., 2020; Qu et al., 2020; Zou et al., 2020); nevertheless, the demands for higher volumetric energy density and cycling stability are still unsatisfied.

Well-dispersed single-crystalline NMC particles have been widely recognized with higher volumetric energy density and superior stability. Firstly, the individual single-crystalline NMC particle holds better structural integrity than the loosely aggregated polycrystalline particle during high-pressure pressing, and therefore higher tap density can be anticipated to achieve higher volumetric energy density (Kim et al., 2018; Zhong et al., 2020). Secondly, the lattice volume expansion/contraction in the single-crystalline NMC is isotropic, which significantly lowers the risk of microcrack evolution compared with the polycrystalline NMC (Liu et al., 2018). Thirdly, the coating of conductive agent is more homogeneous on the well-dispersed single-crystalline particles, which renders better electronic conductivity (Kimijima et al., 2016b). Single-crystalline particles have a smaller surface area for Li insertion/extraction reactions compared with the porous polycrystalline NMC; therefore, a median size of 1–4 um is critical for the single-crystalline NMC to maintain rate capabilities similar to those achieved by conventional polycrystalline NMC (Kimijima et al., 2016a). To date, several techniques, including solid-state sintering (Huang et al., 2011; Zhu et al., 2012; Lin et al., 2015; Wang et al., 2016; Jiang et al., 2017; Fan et al., 2020; Zhong et al., 2020), rheological reaction (Han et al., 2010), and molten flux growth (Kim, 2012; Kimijima et al., 2016a,b; Jiang et al., 2017), have been used to synthesize single-crystalline NMC cathode; equivalent electrochemical performances have been achieved in terms of capacity and rate capability compared with polycrystalline NMC. Moreover, the cycling stability of single-crystalline NMC outperforms the polycrystalline product. Each work had discussed the growth mechanism, but the understandings are mostly based on individual techniques. A deep review and analysis can help draw a more fundamental and universal growth mechanism of the single-crystalline NMC, which would facilitate the knowledge-based improvement of synthesis techniques.

## Growth Mechanism: General Considerations for NMC Cathode

A crystallization process generally comprises of two stages: First, the formation of three-dimensional nuclei from the supersaturated medium (or matrix), and second, the nuclei grows into a larger crystal entity (Sangwal, 2018). In the first stage, supersaturation is a prerequisite for the new phase to nucleate, which can be reached by concentration fluctuation, solvent evaporation, and cooling of the medium. As long as the nuclei's size can exceed the critical value *R* (Mittemeijer, 2010):

$$\begin{array}{l} R=\frac{2\gamma }{\Delta G_{chem}^{v}+\;\Delta G_{strain}^{v}} \end{array}$$

where γ is the interfacial energy per unit (interfacial) area, $\Delta G_{chem}^{v}$ is the energy contribution driving the phase transformation, and $\Delta G_{strain}^{v}$ is the strain energy per unit volume.

The phase change-incurred free energy drop would overcome the increase of surface free energy, and the nuclei would be stable. Otherwise, it would be dissolved into the medium. In the second stage, the mass transportation controls the growth rate of crystals when the mean distance between grains is far enough (Lifshitz et al., 1961). As time passes by, the closed system tends to achieve a minimal energy state by reducing the overall surface energy, where the Ostwald ripening (grain coarsening) process would dominate (Sangwal, 2018).

**Figure 1:**
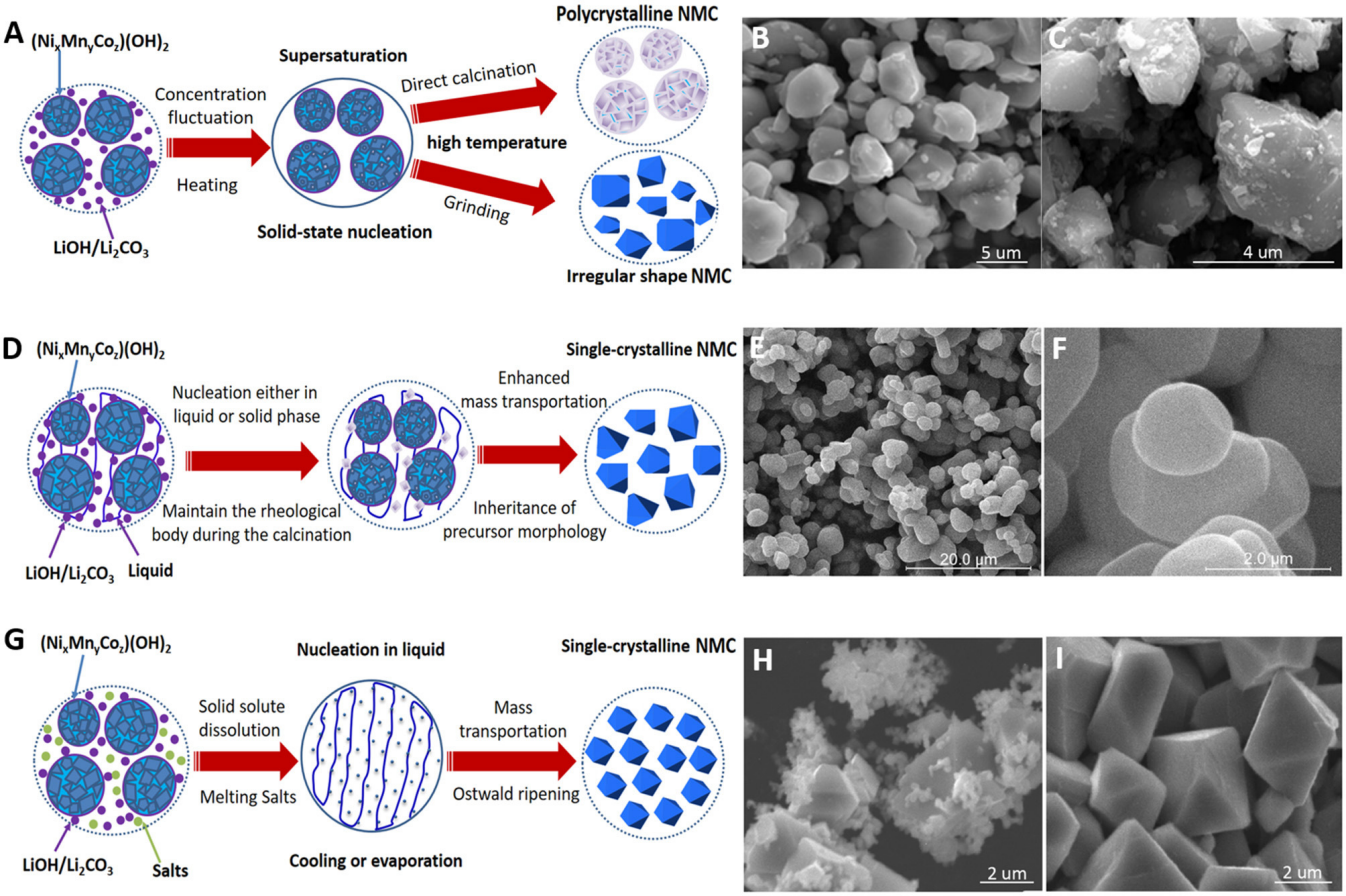
**(A)** Growth mechanism of solid-state reactions. **(B)** Lithium nickel manganese cobalt oxide (NMC) product of multiple calcinations using aggregated precursor prepared by coprecipitation method (Fan et al., 2020). **(C)** NMC product of 900°C calcination using uniformly dispersed precursors prepared by hydrothermal reaction (Wang et al., 2016). **(D)** Growth mechanism of rheological reaction. **(E,F)** Calcination of spherical template in rheological body produces spherical single-crystalline NMC (Han et al., 2010). **(G)** Growth mechanism in molten salt flux. **(H)** Diphasic particle morphology of NMC produced, synthesized in a dilute Na_2_SO_4_ molten flux (20 mol.%). **(I)** Well-dispersed single-crystalline NMC synthesized in concentrated Na_2_SO_4_ molten flux (80 mol.%) (Kimijima et al., 2016a).

The synthesis of NMC cathode usually uses a mixture of transition metal hydroxides/nitrides/sulfates and LiOH/Li_2_CO_3_ as the precursor medium. A minimal heat input above 200°C can trigger enough concentration fluctuation for the nucleation of NMC (Zhu et al., 2012); however, the grain growth is negligible until the calcination temperature reaches the melting point of LiOH/Li_2_CO_3_. In the melts of Li precursors, the mass transportation is accelerated, and so is the crystal growth (Grigorova et al., 2011). When the calcination temperature reaches above 800°C (Zhu et al., 2012; Kong et al., 2019), the slight evaporation of Li_2_O can further facilitate the mass transport and results in significantly accelerated crystal growth since the homogeneous distribution of transition metal ions has usually been achieved by coprecipitation or milling, and the NMC lattice formation mostly relies on oxygen and lithium migration, whereas the phase and the composition of NMC would begin to change when calcined at 900–1,000°C due to vigorous lithium volatilization (Wang et al., 2017; Zhao et al., 2017). To date, many works have been devoted to synthesize single-crystalline NMC cathode and various methods have been developed, which can be generally categorized into solid-state reaction, solid–liquid reaction, and molten flux growth. As illustrated in Figure 1, the distinct differences in nucleation, mass transportation, and growth environment yield differences in agglomeration, size, and shape of NMC product. In the following sections, we will review and discuss the mechanisms and the key factors dominating the growth of single-crystalline NMC cathode in different methods.

### Solid-State Reaction

The most prominent feature of solid-state reaction is that the NMC product can easily inherit the morphology from the solid precursors (Figure 1A). The nucleation of the new phase takes place in the solid precursor medium, and the growth of grains is constrained by the mass transport inside the rigid lattice. As a consequence, most commercial NMC products synthesized through the coprecipitation route inherit the agglomerated polycrystalline spheres from the (Ni_*x*_Mn_*y*_Co_*z*_)(OH)_2_ precipitates. Constrained by the solid precursor medium, it is very hard to obtain well-dispersed single-crystalline NMC particles in single-time calcination. Even under extremely high temperatures over 1,000°C, the greatly enhanced mass transportation between grain boundaries can only yield 500-nm primary particles closely aggregated together (Zhu et al., 2012). Li et al. (2017), Zhong et al. (2020), and Fan et al. (2020) have demonstrated that additional grinding and multiple calcinations can help obtain 1–3-um single-crystalline NMC below 1,000°C (Figure 1B). During heating or calcination, additional grinding can break the agglomeration and expose a large number of fresh surfaces, where the grain boundary diffusion can be greatly enhanced and the energy barrier for crystal growth can be overcome easier. Altering the agglomeration state of the solid precursor medium and elevating the calcination temperature can alleviate agglomeration and improve the crystallinity. Huang et al. (2011) firstly prepared highly porous solid precursors through the violent evaporation of volatile species from a gel, then solid-state calcination between 900 and 950°C produces 500-nm single-crystalline NMC111 particles loosely interconnected with each other. Lin et al. (2015) added H_2_O_2_ during the coprecipitation process, which then decompose and produce a large amount of O_2_ gas bubbles to disperse the precursors, and finally they obtained loosely connected 300-nm NMC111 single crystals after 800°C calcination. Jiang et al. (2017) used the eutectic mixture of LiOH and LiNO_3_ as an alternative Li source for solid-state reaction, and the melting point of the eutectic mixture was below 200°C, which is much lower than that of the conventional LiOH precursor (500°C). As a result of the sufficient and fast lithium diffusion, they successfully synthesized NMC111 particles of about 700 nm after 900°C calcination, and the aggregation is alleviated. In addition to the coprecipitation method, Wang et al. (2016) firstly prepared a well-dispersed M(C_2_O_4_)·2H_2_O (M = Ni, Mn, and Co) mixture through hydrothermal reaction and then ground them with LiNO_3_. Finally, they obtained NMC622 single crystals of about 1–2 um, and the aggregation is very slight after 800–900°C calcinations (Figure 1C).

### Solid–Liquid Rheological Reaction

The growth of a single crystal is easier in solid–liquid rheological reaction than in solid-state calcination, which has been successfully used to synthesize single-crystal Ni(Sal)2·4H_2_O [Sal = C_6_H_4_OH(CO)_2_] (Wang et al., 2003) and Cu(C_6_H_5_CO_2_)3H (Zhao et al., 2011). The rheological body is a uniform mixture of solid precursors and liquid substances (Tang et al., 2002), in which mass transportation is certainly easier than in pure solid state and phase nucleation could be either in the solid phase or in the supersaturated region of the liquid phase (Figure 1D). Maintaining the rheological body during calcination is critical to obtain the single-crystalline NMC particles. Unfortunately, most liquid substances used in rheological reactions cannot exist at temperatures higher than 300°C; the rhetorical body in the mixing or preheating process can only improve the element distribution, but the final product still inherits the polycrystalline and the agglomeration nature of the solid precursors (Xiao et al., 2004; Li et al., 2008, 2014, 2016; Shi et al., 2009, 2014). Han et al. used the conventional rheological reaction to uniformly insert the very fine nanoscale NiO and Co_3_O_4_ into spherical amorphous MnO_2_ at the first step; interestingly, during 950°C calcination, the assembled nanostructured precursor easily melted and formed a glass-like rheological body, as displayed in Figures 1E,F. Eventually, well-dispersed single-crystalline NMC111 particles of about 2–4 um were successfully obtained (Han et al., 2010).

### Crystal Growth From Molten Salt Flux

Crystal growth in molten salt flux has several advantages, including (1) facilely obtaining multicomponent inorganic crystals at temperatures far below the melting point of the precursor solute, (2) crystal growth is free of the rigid mechanical constraint of the solid precursors, and (3) the salts are generally cheap and environmentally friendly. As illustrated in Figure 1G, firstly, the precursor solutes are dissolved in the molten salts and then reach supersaturation, through either flux evaporation or cooling, to enable nucleation. After nucleation, the crystal growth in the molten flux is subject to mass transportation and the Ostwald ripening process. The holding temperature, solute concentration, and reaction time are key factors that influence the single-crystalline NMC products in the flux growth method (Wang et al., 2020a).

First of all, the holding temperature should be high enough to melt the salt and enable the precursor solute to dissolve into the flux. Kim (2012) tried to dissolve the coprecipitated (Ni_0.8_Mn_0.1_Co_0.1_)(OH)_2_ into NaCl (melting point, 800°C) and KCl (melting point, 770°C) at 800°C. The fully melted KCl salt yields well-separated single-crystalline NMC811 particles, while the partially melted NaCl salts generated NMC811 particles that inherit the agglomeration nature of the (Ni_0.8_Mn_0.1_Co_0.1_) (OH)_2_ precursor. Generally, the higher holding temperature can give rise to a lower viscosity of the melt; both the solubility and the mobility of solute components can be improved by a higher holding temperature, which will result in a larger size of the single-crystalline particles (Teshima et al., 2011; Kim, 2012; Kimijima et al., 2016b). It is worth to note that the gravimetric capacity of the single-crystalline NMC could be seriously dampened when the holding temperature exceeds 900°C (Kim, 2012; Kimijima et al., 2016b) since a large amount of Li is volatilized.

In a flux with very low solute concentration, where all solutes are fully dissolved, supersaturation and nucleation are almost randomly achieved, and the subsequent crystal growth is independent with each other. Under such condition, a diphasic particle morphology (Figure 1H) of the final product is commonly seen, in which the micron-sized crystals with well-developed facets coexist with nanoscale crystals possessing irregular shapes (Teshima et al., 2011; Kimijima et al., 2016a). When the solute is partially dissolved, the remaining solid solute would play as sources for supplying reactive mass into the salt flux by diffusion. Supersaturation is easier to reach in the flux region adjacent to the undissolved solute to enable nucleation. In such a scenario, the solute concentration of the mixture can be considered as the average distance among these undissolved solid solute particles. Increasing the solute concentration would decrease the average distance among the sources for supplying mass, which would facilitate a fast and sufficient transportation of reactive mass to the growing crystals. As a result, most flux growth works have reported that the particle size of single-crystalline NMC increases with increasing solute concentration (Kim, 2012; Kimijima et al., 2016a,b), as compared in Figures 1H,I.

The crystal growth in the molten flux inevitably introduces the additional solid/liquid interfacial energy, which varies with the interface area. Thermodynamically, the system tends to reach a more stable state by decreasing the interfacial energy, and this could be achieved by increasing the average size of the crystals (Ostwald ripening) (Sangwal, 2018). With increasing reaction time, the smaller crystals would dissolve into the flux to feed the larger crystals and make them grow larger, and eventually the crystal size distribution can be improved (Kimijima et al., 2016a,b).

## Manipulation of Morphology

Manipulating the shape and the crystal orientation of cathode particles could help gain advantages in both tap density and electrochemical performances. As described above, the nucleation and the growth of single-crystalline NMC generally take place in a less-constrained environment, where several measures can be adopted in different stages to manipulate the morphology.

### Control of Shape

In the growth process which is closely associated with solid precursors, the rigid solid structure and the stagnant mass transportation make it hard for the final product to deviate from the shape of the precursors (Huang et al., 2011; Wang et al., 2016; Zhong et al., 2020). On the other hand, the shape inheritance from solid precursors can be utilized as templates for the final products' shape control. Han et al. (2010) have used the spherical amorphous MnO_2_ particles as growth templates in a rheological reaction to synthesize single-crystalline NMC111. In the glass-like mixture (LiOH, NiO, and Co_3_O_4_) of oxide melt and amorphous MnO_2_, the mass transportation and the ionic rearrangement are constrained by the solid sphere of MnO_2_, of which the spherical shape also helps minimize the surface energy during growth. Eventually, spherical single-crystalline NMC111 particles were successfully synthesized. Jiang et al. (2017) firstly synthesized polygon (Ni_0.29_Mn_0.33_Co_0.38_)(OH)_2_ nanoplates with only 20-nm thickness and then calcined in a LiOH–LiNO_3_ mixture melt at 900°C. During the solid–liquid reaction, lithium and oxygen in the melt can easily attach and react on the surfaces of the nanoplates; by rapid diffusion of lithium and oxygen, the melt glue and assemble these polygon nanoplates along their axial direction, and eventually, polygon-shaped single-crystalline NMC with thickness of 200–500 nm have been obtained.

In the case of growth in liquid medium such as molten salt flux, the surface atoms of the infant nucleus are in high ratio and loosely bonded, which are easily influenced by subtle mechanical or thermal perturbation in the medium. The initial morphologies of the infant nucleus are random and less constrained by their lattice. If the NMC nucleus grows unimpeded, the idiomorphic growth of crystal ultimately acquires a well-defined octahedral shape, and this is achieved by sufficient mass transportation from the supersaturated medium and efficient mass migration on the crystal surfaces.

Takeshi et al. (Kimijima et al., 2016a) reported that single-crystalline NMC111 particles grown in molten Na_2_SO_4_ flux exhibit an octahedral shape with a flat surface, but which, when grown in the Li_2_SO_4_ flux, exhibit a blurred shape and a smaller size. The difference can be ascribed to the higher solubility of the Li_2_SO_4_ flux, which decreases the supersaturation degree of the medium and cannot drive sufficient precipitation from the flux to attach on the crystals' surfaces. Single-crystalline NMC811 particles grown in KCl exhibit an isotropic shape, whereas the octahedral shape with well-developed (003) and (11–1) facets is acquired in the NaCl flux. Kim proposed that the KCl flux may reduce the surface energy of the facet (Kim, 2012), but the exact influence of the KCl and the NaCl fluxes on the surface energy of the NMC crystal has yet to be clarified. We have noticed that the KCl flux can dissolve the solute precursor easier than the NaCl flux in the work of Kim (2012), which implies that the solubility of the KCl flux is higher than that of the NaCl flux. Moreover, the NMC811 particles (<2 μm) grown in the KCl flux are much smaller than those (>5 μm) in the NaCl flux. All of these imply a less-sufficient mass transportation in the KCl flux due to higher solubility and less supersaturation.

It can be concluded that the morphology of single-crystalline NMC products is mostly dominated by their growth environment. The solid-state phase transition is rigidly constrained by a hard template, and the morphology inheritance from the precursor is inevitable unless extremely high temperatures significantly break the rigid constraint of solids. The growth in liquid or liquid/solid mixture is freer of mechanical constraint. Therefore, the growth of well-defined crystal planes would be easier. Without the rigid constraint of the solid precursor, manipulation of the morphology is feasible by altering the chemical or the thermal conditions. Whatever the growth environment is, the particle size of the final product is mostly dominated by the mass transportation rate in a limited growth time. Generally, a fast mass transportation, which is enabled by either a high temperature or a steep concentration gradient or a dramatic variation of solubility, can facilitate crystal growth or grains to coalesce to yield large primary particles.

### Control of Facet

Owing to the hexagonal layered structure, the NMC crystal can enable fast lithium transportation through the {010} facets while impeding lithium migration through the {001} facets. Obviously, the highly exposed {010} facets of the NMC cathode would give rise to enhanced rate capabilities. Unfortunately, the surface energy of the {010} facets is higher than that of the {001} facets, which leads to a higher growth rate and eventually the disappearance of the {010} facets in an uncontrolled growth process. In the recent decade, many works have successfully synthesized NMC products with highly exposed {010} facets through using a surface capping agent, adjusting the pH value, and modifying the tank reactor (Wei et al., 2010; Fu et al., 2013; Chen et al., 2014; Hua et al., 2017; Yu et al., 2017; Ju et al., 2018; Su et al., 2018; Zhou et al., 2018; Dong et al., 2019; Xiang et al., 2019). Dramatically increasing the {010} facets generally causes a serious agglomeration, owing to the large surface area. Although a porous or a hierarchical structure can help expose the {010} facets of the agglomerated secondary particles, it may further decrease the tap density of the NMC product. The synthesis of well-dispersed single-crystalline NMC with preferred facets still remains a great challenge. There are some works that can give clues for tuning the facets of single-crystalline NMC. Fu et al. (2013) added a large amount of polyvinylpyrrolidone during the coprecipitation process, creating a viscous environment which dramatically slows the nucleation rate. The limited number of nuclei adsorbed by PVP grows into nanoplates in the length of 200 nm in the solution. They further tuned the calcination temperature to increase the thickness, and eventually, single-crystalline NMC111 with exposed {010} facets are obtained. Kim (2012) and Takeshi et al. (Kimijima et al., 2016a) all have pointed out that the Na^+^ ions in the molten salt flux can help the NMC crystallites expose more {003}, {101}, and {111} facets. Since Na^+^ ions were not found to incorporate into the NMC lattice, it was believed that the surface energy or the surface mass migration conditions can be influenced by Na^+^ ions in flux.

## Discussion and Perspective

As discussed above, the main purpose of developing a single-crystalline NMC cathode is to achieve superior volumetric energy density and cycling stability. During the development progress, a median size of 1–4 um is found critical for a single-crystalline NMC cathode to maintain competitive rate capabilities. Solid-state reaction with additional grinding and calcination can facilely produce single-crystalline NMC, but the particles' shape is irregular and mild agglomeration still remains. Molten-flux-based methods have exhibited superiority in the synthesis of well-dispersed single-crystalline NMC cathode, including flexibility of the precursor's choice and easy to acquire micron-meter-sized well-crystallized primary particles. In addition, the particle size distribution is tunable in the Ostwald ripening process by altering the reaction temperature and the holding time, whereas the manipulation of crystal shape and the preferred facets is still premature in molten-flux-based growth. The rheological reaction exhibits a promising synergic effect of rapid mass transportation in liquid and morphology inheritance from solid precursors. However, the speculation on the details of the solid–liquid reaction process still lacks complete physical chemistry insights.

In view of the capital cost for industrial production, the solid-state synthetic route requires almost no facility investment to produce single-crystalline NMC. Grinding, multiple calcinations, and a well-dispersed precursor can be realized in the current facilities. However, both the rheological reaction and the molten flux growth route require modifications on the conventional tunnel furnace to prevent an explosive spillover of hot liquid and to more accurately control the cooling rate. In view of energy consumption, the molten flux growth can yield micron-sized single-crystalline NMC in one-step calcination below 900°C, whereas the solid-state synthetic route generally requires multiple steps, with a final heating above 900°C. In view of product quality, both the molten flux growth and the rheological reaction are fascinating since the fast mass transportation and the less-constrained growth environment can produce well-dispersed particles with uniform size and morphology. In addition, low-temperature calcination (<900°C) can prevent significant Li/O loss.

The fully controllable synthesis of single-crystalline NMC is dependent on deep understandings of phase nucleation, mass transportation, and surface energy variations. In the past, the closed holder containing the liquid/solid reactive materials at high temperatures has hidden the details of the growth process and left puzzles of morphology variation. The recent advances on *in situ* transmission electron microscopy and neutron diffraction methods can help interrogate the key factors that influence single-crystalline NMC growth (Shoemaker et al., 2014; Vasquez et al., 2019; Wang et al., 2020a), and theoretical computations of surface energies and their variations in different environments would guide an efficient manipulation of crystal morphology.

## Author Contributions

TW searched the relevant literatures, draw the figure draft, and acquired rights/permissions for SEM pictures. HW proposed the topic, guided the analysis, and wrote the whole manuscript. All authors participated in the analysis on previous work and discussed the growth mechanisms.

## Conflict of Interest

The authors declare that the research was conducted in the absence of any commercial or financial relationships that could be construed as a potential conflict of interest.
